# Telemedicine Use in Rural Native American Communities in the Era of the ACA: a Systematic Literature Review

**DOI:** 10.1007/s10916-016-0503-8

**Published:** 2016-04-27

**Authors:** Clemens Scott Kruse, Shelby Bouffard, Michael Dougherty, Jenna Stewart Parro

**Affiliations:** School of Health Administration, Texas State University – San Marcos, 601 University Drive, San Marcos, TX 78666 USA

**Keywords:** Native Americans (Indians, North American), Telemedicine, Telehealth, Patient Protection and Affordable Care Act, Rural population

## Abstract

Native American communities face serious health disparities and, living in rural areas, often lack regular access to healthcare services as compared to other Americans. Since the early 1970’s, telecommunication technology has been explored as a means to address the cost and quality of, as well as access to, healthcare on rural reservations. This systematic review seeks to explore the use of telemedicine in rural Native American communities using the framework of cost, quality, and access as promulgated by the Affordable Care Act of 2010 and urge additional legislation to increase its use in this vulnerable population. As a systematic literature review, this study analyzes 15 peer-reviewed articles from four databases using the themes of cost, quality, and access. The theme of access was referenced most frequently in the reviewed literature, indicating that access to healthcare may be the biggest obstacle facing widespread adoption of telemedicine programs on rural Native American reservations. The use of telemedicine mitigates the costs of healthcare, which impede access to high-quality care delivery and, in some cases, deters prospective patients from accessing healthcare at all. Telemedicine offers rural Native American communities a means of accessing healthcare without incurring high costs. With attention to reimbursement policies, educational services, technological infrastructure, and culturally competent care, telemedicine has the potential to decrease costs, increase quality, and increase access to healthcare for rural Native American patients. While challenges facing the implementation of telemedicine programs exist, there is great potential for it to improve healthcare delivery in rural Native American communities. Public policy that increases funding for programs that help to expand access to healthcare for Native Americans will improve outcomes because of the increase in access.

## Introduction

Innovations in technology improve healthcare for disparate groups of people in the United States. One such innovation, telemedicine, promises access to care for those even in the remotest areas. Telemedicine has been considered a potential method of healthcare delivery for both Native American and Alaskan Inuit reservations through the Indian Health System (IHS) since the early 1970s [1]. The populations served by the IHS demonstrate the highest prevalence of type 2 diabetes in the world, fight cardiovascular disease as the leading cause of mortality, demonstrate the highest rate of substance abuse and dependence in the nation, and possess the greatest mortality rates from alcoholism, tuberculosis, accidents, homicide, and suicide than all other Americans [2]. The IHS is a nationwide network of hospitals and clinics that services 1.9 million American Indians and Alaskan Natives who belong to some 564 tribes across 35 states with only $4.4 billion [3]. The IHS must spread its resources much more thin than the private delivery system that exists in the rest of the US. Some services are not provided from within its own system. Because the IHS does not keep pediatricians on staff in the state of Montana, it contracts with other health organizations such as St. Vincent Healthcare and delivers pediatric services through telemedicine appointments [4]. In order to improve the overall health of the population served by the IHS without a significant increase in the network itself, other means or modalities of care must be exploited. Telemedicine offers such a change. Telemedicine is recognized as a modality of delivery of care to many populations including medically underserved areas [5]. While the use of telemedicine has expanded since its introduction in the IHS in the early 1970s, there are still significant challenges and threats to widespread implementation [6].

### Identification and definition of key terms

For the purpose of this paper, we use the World Health Organization’s (WHO) definition of telemedicine:

The delivery of health care services, where distance is a critical factor, by all healthcare professionals using information and communication technologies for the exchange of valid information for diagnosis, treatment and prevention of disease and injuries, research and evaluation, and for the continuing education of health care providers, all in the interests of advancing the health of individuals and their communities [7].

Accordingly, while some authors and organizations interpret telemedicine and telehealth differently, the WHO definition remains the standard and, throughout this paper, we will not distinguish between the two. Furthermore, such terms are used broadly to refer to many different technologies (e.g. remote monitoring, store and forward technologies, videoconferencing) and fit the aim of this study to synthesize the literature pertaining to these individual technologies into one place.

Within this report, several terms are used to refer to places where Native American communities exist, including: Tribal lands, rural Native American communities, Native American reservations, and reservations. Further, within this research, the term Native American will be used ubiquitously to refer to indigenous, or first nations, groups in the United States, and peoples in the United States who live on a nationally recognized reservation. Out of respect for the people and the culture, we refrain from using the contentious and problematic terms “American Indian” or “Indian” in all regards, except with respect to search terms or formal names of agencies or organizations (e.g., Indian Health Service). Other phrases that may be used interchangeably to refer to Native Americans include more widely accepted terms such as indigenous groups, first nations, and native peoples.

This work organizes data based on cost, quality, and access indicators, which were drawn from the Patient Protection and Affordable Care Act of 2010 (ACA). This federal policy passed with the intent of expanding Americans’ access to healthcare services while also reducing costs of care and rewarding delivery of high quality care. This study uses the three indicators to determine the efficacy of telecommunications technology in rural Native American communities and to discuss potential solutions to addressing challenges of telemedicine program implementation.

This systematic literature review seeks to accomplish two things. First, it attempts to identify areas where the use of telemedicine will have a positive impact on rural, inaccessible, and poverty-stricken places in America. Second, it works to consolidate and streamline information to address potential solutions that will encourage broader adoption of telecommunication technology for the provision of medical care.

### Research questions

The questions we seek to answer in conducting this systematic review are:

- R_q1_ – What impact does telemedicine have on cost, quality, and access for Native American rural communities?
- R_q2_ – What potential solutions to existing problems arise from the literature?
- R_q3_ – How can cost, quality, and access be improved to better accommodate Native American rural community health?

## Methods

### Eligibility criteria

This section outlines the protocol used to qualify articles for analysis and to explain how multiple reviewers were trained to decrease selection bias and increase inter-rater reliability. The format of this systematic literature review follows content as outlined in the Preferred Reporting Items for Systemaitc Reviews and Meta-Analyses (PRISMA, 2009). In an effort to ensure that the information used as the basis for this study is accurate and pertinent, we established several eligibility criteria. All articles considered for inclusion in this study were peer reviewed articles or from academic journals. Only articles that were published since the beginning of 2005 were considered for inclusion in this study because we thought 5 years prior to and 5 years after the ACA would be sufficient to capture applicable information. Additionally, articles needed to be published in English in order to be considered for inclusion in this study. This manuscript began as directed research in a master level program. Part of the class included research methods and proper searches in quality journals. Weekly meetings on this research were conducted to refine the search strategy and ensure we were all looking for consistent content.

### Information sources & search

**Fig. 1:**
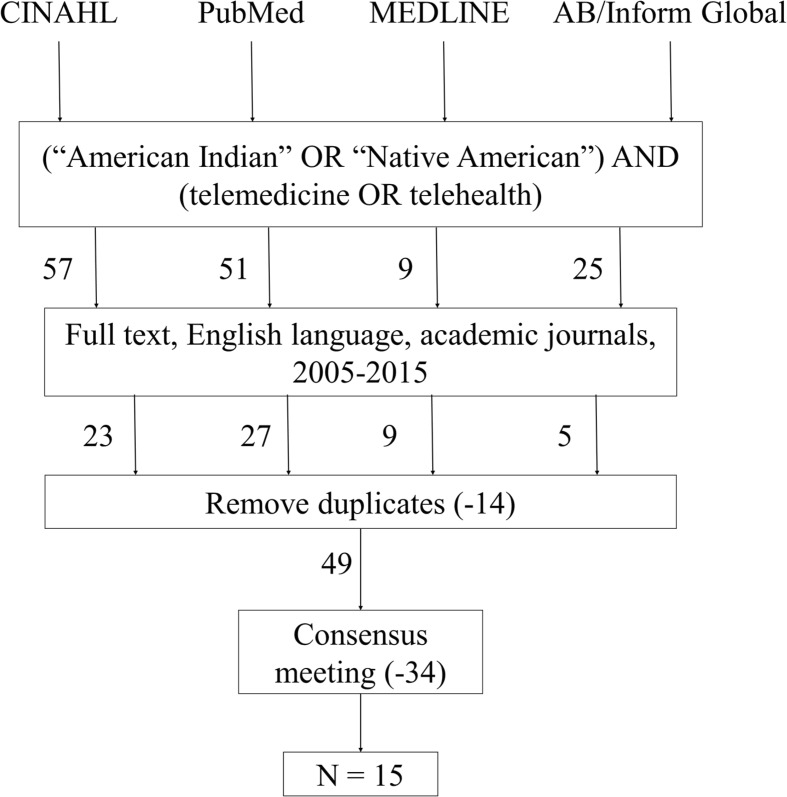
Summary of search methods, inclusion and exclusion criteria, and article selection

We searched four databases for this research: CINAHL, PubMed, MEDLINE, and AB/Inform Global. CINAHL was chosen because of its exhaustive search of nursing, health administration, health information management, long-term care, health services physical therapy, respiratory care, radiation therapy, communication disorders, clinical laboratory science, and other allied health disciplines from 1937 to present. PubMed and MEDLINE were chosen because of their premiere status as international indices of health-related sciences. Although MEDLINE is automatically searched by both CINAHL and PubMed, we also searched it separately to ensure we captured all applicable articles. AB/Inform Global was chosen as a portal to ProQuest, which, among other sources, is an index of dissertations and theses. Figure 1 illustrates the selection process, including criteria for inclusion and exclusion. We used Boolean search terms to increase specificity and ensure consistent searches across disparate databases. The subject terms “Native American,” “American Indian,” and “indigenous” were used in combination with “telemedicine” and “telehealth” in a variety of searches within the databases, resulting in 62 total articles. After adjusting for 14 duplicates, 48 unique articles remained. In addition to the exclusion criteria, each article was reviewed for pertinence to the topic of telemedicine use for the provision of healthcare to Native American reservations and consensus meetings were held with all reviewers. Articles were excluded if the content did not considerably focus on this topic. There was one exception to these criteria due mainly to the background information provided by this article [8].

### Study selection

Following the selection of articles that met eligibility criteria, the articles underwent a subjective review process for inclusion that considered the pertinence of the data and the value to our review objectives. Each author of this study reviewed at least eight of the articles subjectively to determine eligibility for inclusion, resulting in a recommendation to either include or exclude each article by the reviewing author. Consensus meetings were held to share notes. In instances in which the two reviewers agreed in their recommendations (either to include or to exclude), the recommendation was followed. In instances in which the two reviewers differed in their recommendations, the remaining reviewer evaluated the article and made the final recommendation (*n* = 15).

### Risk of bias

During the selection process, the reviewers screened articles by reading only the abstracts of the articles. The subjective assessment of an article’s pertinence by the reviewer introduced a source of bias to this study. However, we attempted to mitigate this bias by requiring that two reviewers agree on an article’s inclusion and that they share their notes in a consensus meeting.

Furthermore, because of the limited scope of the subject of this review, we found that several of the articles shared authors. Six authors included in the review were involved in at least two of seven of the articles included in this study. We recognize that this limits the number of perspectives published by the articles the review, however, because of the limited research that has historically been conducted on this topic, we felt it was necessary to include these articles.

## Results

### Analysis of results

As each researcher verified the inclusion of articles for this study, references to the three variables explored in this study were counted within each article, and the articles were ranked against cost, quality, and access as shown in Table 1. These rankings were then incorporated into a statistical analysis, which demonstrates that the greatest area of opportunity for the use of telemedicine is in improving access to healthcare. While this idea seems intuitive, the data from the literature review reinforces this idea. When ranked in terms of pertinence to cost, quality, and access, access was ranked in ten articles as the primary subject matter of the article; quality was ranked in eight articles to be the second most pertinent subject, and cost was ranked in nine articles to be the least applicable subject.

**Table 1:** An illustration of the reviewed articles and their respective cost, quality, and access thematic rankings

Article title	Cost rank	Quality rank	Access rank
Telehealth and Indian healthcare: Moving to scale and sustainability [1]	2	3	1
Telemedicine: What it is, where it came from, and where it will go [8]	1	3	2
Indian Health Service Innovations have helped reduce health disparities affecting American Indian and Alaska Native people [9]	3	1	2
Satisfaction with telehealth for cancer support groups in rural American Indian and Alaska Native communities [10]	3	2	1
Enhancing access to cancer education for rural healthcare providers via telehealth [11]	3	2	1
Expanding the walls of the health care encounter: Support and outcomes for patients online [12]	3	2	1
Role of telehealth/videoconferencing in managing cancer pain in rural American Indian communities [13]	3	2	1
Innovation in Indian healthcare: Using health information technology to achieve health equity for American Indian and Alaska Native populations [14]	3	2	1
The diffusion of telehealth in rural American Indian communities: A retrospective survey of key stakeholders [15]	3	2	1
Patient and provider perspectives on using telemedicine for chronic disease management among Native Hawaiian and Alaska Native people [16]	3	2	1
Telepsychiatry services at a tribally run behavioral health clinic [17]	2	3	1
Reaching rural communities with culturally appropriate care: A model for adapting remote monitoring to American Indian veterans with posttraumatic stress disorder [18]	3	2	1
Comparing the effectiveness of telemedicine and traditional surveillance in providing diabetic retinopathy screening examinations: a randomized controlled trial [19]	3	2	1
Characteristics of telemental health service use by American Indian Veterans [20]	3	2	1
Acceptability of telepsychiatry in American Indians [21]	3	2	1

### Study characteristics

What makes this study unique is its focus on the indicators of cost, quality, and access, laid out by the ACA, as measures of strength in using telemedicine in rural Native American communities. Our analysis demonstrates that telemedicine has the greatest impact on access. Each of the three dimensions of the ACA is explored separately, as each uniquely influences telehealth adoption in Native American populations. While these characteristics of healthcare serve as positive distinguishers, the true power of this study comes from the consolidation of the positive effects on cost, quality, and access related to health outcomes since the incorporation of telemedicine in the provision of healthcare to rural Native American communities began.

## Discussion

### Summary of evidence for cost, quality, and access indicators

#### Cost

In general, rural Native American populations incur significant costs, both economical and social, to receive health care. The literature, which explores these costs, indicates that such costs are incurred via travel, time, pain, and care delivery. One study found that for every dollar spent in telehealth, $11.50 was saved in travel and child-care expenses and without any decrease in quality (14).

- *Travel*. Studies cited travel costs as a serious problem for Native American healthcare. In order to receive specialty care (which is often unfunded in Indian Health Service (IHS) facilities), those living on reservations must travel great distances, as reservations are typically geographically isolated [9]. One study examining access to cancer support groups noted that trips often require between 2 to 5 h of travel each way, with travel costs alone ranging from $50 to $200 [10]. For Native American communities in Alaska, for example, the vast distance between the reservation and the nearest medical facility compares to the distance between Chicago and New York, costing patients $100 to $1200 for travel to seek care [10].
  Telemedicine arises as a solution to reduce travel costs incurred by these populations by eliminating the need for extensive patient travel, thereby reducing costs while also increasing access, regardless of geographic position. Telemedicine also eliminates a need for providers to travel; one study of access to cancer education for rural providers noted that travel costs, lack of financial resources in general, and time away from work all prohibit providers from attending educational conferences in more urban settings [11]. Real-time interactions for both patients and providers via telemedicine eliminate these travel costs [11].
- *Time*. An additional vector for costs incurred by Native American patients seeking healthcare is time. Time spent away from work in order to travel represents a potential opportunity cost in the form of lost wages. Further, many patients must also seek childcare in order to travel [10]. Though indigenous tribes of North America vary widely in beliefs and practices, women often fill the role of caretakers; thus, the need for childcare may disproportionately impact the health of Native American women, who may be prohibited from seeking healthcare due to childcare constraints.
  Time costs, too, are addressed by the use of telemedicine. A traditional interaction between a patient and a provider lasts approximately 15 to 30 min [12]. However, one study demonstrated that telehealth reduces the need for this much time because patients can simply email their providers [12]. This study also suggested that remote monitoring systems and Internet access to a patient portal provide a more economical approach to both assessment and monitoring of diabetic patients. Another article [9] noted that the ability to teleconference with a provider asynchronously via messages allowed for IHS clinicians to submit questions to specialists without requiring a scheduled video session because the specialist may reply at his or her convenience.
- *Pain*. Another cost that results from the current healthcare delivery model in Native American populations is pain. Providers who are not culturally competent may react differently to assessments of pain from patients of ethnicities other than that of the provider [9]. This increases the risk of inadequate pain treatment, as only 15 % of physicians working in IHS facilities self-identify as Native American [9, 13]. This untreated or poorly managed physical pain may further result in an emotional pain resulting from receiving inadequate or inappropriate care within the IHS system.
  The social cost of this pain is shared throughout Native American communities. To address both physical and emotional pain, one study demonstrated that telemedicine applications that focus on pain management are feasible [8]. Telemedicine offers a potential solution to both physical and emotional pain management via conferences with culturally competent physicians.
- *Care Delivery*. The typical healthcare facilities available to rural Native American populations offer comprehensive primary care and prescription drug coverage. For specialist care, contract health service funds are limited to $780 million and are based on medical priorities [9]. This funding is inadequate as compared to the $1.98 billion in funding for IHS facilities [9]. Therefore, specialty care is often only available to Native American populations at high costs to the individual patient [9].
  Telemedicine offers a potential solution to lower the costs of delivering care to Native Americans. IHS minimizes costs by reducing the need for contractual specialty care within IHS facilities [9]. In fact, one study estimated that with the use of telemedicine, general health care costs can be reduced by up to $36 billion per year [8]. Additionally, improving productivity and efficiency of providers will lead to reductions in the cost of care delivery [14].
- *Barriers*. Barriers to using telemedicine for cost savings in Native American populations do exist. The cost of technology is cited in many studies as a deterrent to telemedicine [8]. Healthcare for Native American populations is already vastly underfunded; the per capita health care expenditure for the typical United States population is $8,097 yearly, while for the IHS, per capita expenditure on the population who use healthcare is $3,099, suggesting that funding of technology may be a large deterrent to the adoption of telehealth in rural Native American communities [22].

#### Quality

As a key indicator of healthcare quality, several of the articles mentioned satisfaction with services. For the purposes of this literature review, satisfaction with care will be used as one measure of quality. Harder measures such as the actual quality of the teleconferencing equipment will also be used as a metric.

As the literature was reviewed, several key themes stood out. The IHS has achieved significant quality improvements with regard to the health of Native Americans. Native American life expectancy rose from 63.6 to 72.5 years over 22 years [9]. The key driver of this improvement has been the proliferation of telemedicine resources, which improve patient access to specialists. While not necessarily correlative, this increase in life expectancy came at a time immediately following the implementation of telemedicine technology on tribal lands [9]. Finally, the theme of culturally appropriate education seems to be a leading indicator of health quality. While the improvement in health outcomes for Native Americans is a solid measure of the success of the IHS, softer measures such as education via televideo conferencing is generally regarded as high quality among participants [11].

- *Outcomes*. As previously mentioned, health outcomes have improved significantly since the implementation of telemedicine services on tribal lands. This improvement is measured by a decrease in infant mortality rate, decreases in tuberculosis death rates, and a decline in the unintentional injury rates [9]. All of these results have been achieved during the period of time from 1972 to 2004 [9]. Despite these successes, there are still disparities between rates for Americans and those for Native Americans.
  It is important to note, however, that despite challenges in providing care to Native American communities, outcomes continue to improve. The catalyst for such improvements is the implementation of several telecommunications programs, a specific EHR interface and a special diabetes program specifically designed for Native American populations [9].
- *Education*. The key trend in the use of telemedicine services is for educational and support services. One study describes a cancer education series that took place from 2008 to 2009 that was positively received by participants [11]. In this study, satisfaction scores with the telehealth education services were recorded at 3.6 on a 4-point Likert scale [11]. Despite the positive reception of the educational services, some problems were noted, with the most identified technical issue being audio lag.
  The use of support groups for dealing with the psychological effects of cancer is a proven technique to help cancer survivors [11]. Disparities exist, however, in the delivery of this counseling in a culturally competent manner and to populations in rural settings [10]. One study indicated that telehealth technology can provide this service to rural Native Americans effectively and with high satisfaction rates [10]. Among patients who participated in telehealth support groups, this service experienced a satisfaction rating of 4.59 points out of a possible 5 points on a Likert-style scale [10].
  Telemedicine can also be used to help educate patients in proper self-care techniques, such as in the field of pain management. One study demonstrated that satisfaction rates of 3.35 points out of 5 points on a Likert scale could be achieved with this type of service [8].

#### Access

The Indian Health Service provides health care for an estimated 59 % of Native Americans [22]. As an early adopter of telemedicine technology, the IHS has long sought to improve Native American access to healthcare through remote technology. The Arizona Telemedicine Project was initiated in 1973 to provide health services to rural Hopi, Navajo, Papago, San Carlos Apache, and White Mountain Apache reservation communities [23]. Despite the efficacy demonstrated by the project, telemedicine still faces significant barriers to adoption. The literature revealed two critical themes pertaining to Native American access to healthcare through telemedicine: infrastructural barriers and acceptance.

- *Infrastructural barriers*. Perhaps the most significant barriers preventing widespread adoption of telemedicine in rural Native American populations are infrastructural. Often, Native reservations are geographically isolated, located hours away from urban communities and hospitals [8, 15]. This isolation deters health care professionals such that the IHS physician vacancy rate hovers around 20 % annually [9]. With little access to care on reservations, Native Americans are forced to travel long distances to see a doctor. Furthermore, even when Native Americans have an opportunity to visit an IHS physician, there is still little access to specialized care. Such limited physician options are a problem especially for those suffering with chronic conditions like cancer, kidney disease, or diabetes that generally require more specialized treatment. As previously discussed, telemedicine technologies have the potential to offer Native American communities access to specialized services, support groups, and mental health professionals [10].
  Geographic remoteness, coupled with poor economic conditions, also results in a lack of technological infrastructure. Many reservation communities have inadequate structure or resources for regular telephone or Internet access [11]. The scarcity of these technologies means that access to telecommunications can be a challenge. Further, where telecommunication technology does exist, there can be technical barriers preventing access to care. In one study, providers using telehealth technologies to treat Native American patients cited frustrations with the usability of technology itself as well as lack of staff knowledge of how to operate the tools [16].
- *Acceptance*. Another factor influencing Native American access to care is the acceptance of telehealth technologies by both the patient and provider. Resistance to change, where it exists, is a powerful barrier to access [14]. According to some patients and providers, contact via telecommunications lacks personal connection, limits interaction, and inhibits a sincere patient-provider relationship [16]. As a result, there can be limited acceptance of telehealth technologies on both sides. Where there is acceptance, however, patient access to the provider can be increased and health outcomes are improved. One study among Native Americans with diabetes showed that use of patient-provider email and health portal activity is an effective means of chronic disease management [12]. Another study in indigenous behavioral health demonstrated that use and acceptance of telepsychiatry increased patient access by a factor of six [16].
  A frequently cited need in the selected articles was the necessity for culturally competent and appropriate care. As previously stated, only 15 % of IHS physicians identify as Native American; as such, appropriate cultural communication can be a significant barrier to care [9]. One study among Native veterans with post-traumatic stress disorder identified a model for adapting mainstream healthcare models to better suit Native American communities [18]. By modifying the telehealth model to include components like traditional healing and spirituality, the care became more accessible and culturally acceptable for the suffering Native American veterans [18].

### Proposed solutions to address barriers to adoption

While it is useful to examine the literature to understand the failures and successes of telemedicine, it is also important to synthesize evidence and consider potential solutions to the cited problems that can be transformed into health policy and further research. Such solutions are explored again using the cost, quality, and access model, as each of these themes present different solutions.

#### Cost

As payments for IHS resemble the private sector, reimbursing adequately for telemedicine services is a potential solution to some of the technology costs of telehealth. By expanding telemedicine reimbursement policies for culturally sensitive services, costs of acquiring the telehealth technology may not be as detrimental to populations on reservations who can potentially benefit from this type of service [1].

Another potential solution is the use of a portable system which leverages technology to achieve health equity for Native Americans [14]. A portable system containing a smaller and less expensive camera is far less expensive to develop than larger, more intricate videoconferencing technology, yet still extends care to remote communities and makes teleconferences more practical in small communities. This article showed that the delivery of a portable system to 14 remote, small communities in North Carolina and Alaska has been successful in extending care to patients in these areas [14]. If this idea can be built upon and expanded, the cost of acquiring and implementing proper technology required for telehealth can be substantially lowered, making the system more appealing and attainable for use in Native American populations.

#### Quality

While it is true that quality of care has improved dramatically for Native Americans living in rural communities, the fact remains that significant health disparities still exist between Native Americans and Americans. This issue can be rectified by implementing new policies focused on rural Native American communities and by providing continuing education services. The quality aspects of healthcare that seem to provide the most significant benefit to Native Americans are educational in nature. Despite the programs that IHS has implemented, which have improved quality of care, more can be done. Several of the articles [10, 11, 13] evaluated for this literature review evaluated the impact of temporary programs. These programs demonstrated a significant level of satisfaction among participants. Making programs such as those that help cancer survivors meet, or that help cancer patients manage pain permanent would be an effective step to continue to drive the quest for improved quality.

#### Access

In regard to increasing Native American access to care via telemedicine services, one of the most critical elements to focus on is access to Internet. In 2014, one study suggested that only 10 % of tribal lands have broadband Internet access [24]. Further, in areas where Internet does exist, the communities do not always understand how to use the associated technologies [14]. Therefore, a multifaceted approach to bringing telemedicine programs to Native American reservations includes ensuring the appropriate infrastructure is in place as well as teaching communities how to use the technology and providing technological support. These improvements also must be balanced with cultural views towards technology.

Other structural barriers impeding Native American access to telehealth technologies include access to comprehensive health insurance and education. The Affordable Care Act not only permanently extended the Indian Health Care Improvement Act but also provides for expanded Native access to Medicaid and Medicare [25]. This expansion is important and should not be underestimated, as it allows for impoverished and elder Native Americans to gain access to health insurance. More insured people means the potential for more reimbursement, which could stimulate the growth of telemedicine on rural reservations as long as reimbursement for telemedicine services increases.

To be insured, however, takes significant effort on behalf of the individual. Education, an ever-important piece of any solution, must play a role in order for there to be a significant change in insured rates on reservations. Health providers, including health administrators, physicians, nurses, and technologists, must educate communities on the intricacies of health insurance and its importance. One way to do this might be to host a town-hall community meeting discussing health insurance and addressing questions or concerns that community members might have.

Perhaps the most important part of a multifaceted approach to promoting access and influencing quality to telemedicine on reservations, however, is the inclusion of culturally appropriate telecommunications. Two articles explicitly discuss the need for alternative models of care [15, 18], and one article in particular provides an excellent model for cultural adaptation of telehealth, which should be explored on a greater scale [18]. The simple action of gathering input from local communities, leading to the process of cocreation, has the potential to transform traditional models of telehealth into models more appropriate for and accessible to the Native American patient [18]. Important modifications for a telehealth encounter might include a family-based visit, references to spirituality, community education sessions, and discussion of specific information regarding the health problems and needs of the reservation as a whole (e.g., substance abuse, diabetes, obesity, etc.).

### Limitations of the study

There are multiple limitations to this study. First, we used a ranking system to determine the prevalence of cost, quality, and access in each article. Most articles displayed a common theme of access, although cost and quality were mentioned throughout the literature, which is why these articles were chosen. As access is the most common theme, a limitation on the cost and quality portions of this paper result.

Another limitation of this study is that limited data exists to demonstrate how effective medical care is when provided via telemedicine. There is not a direct link to the improvement in quality of healthcare to the expansion of telemedicine use on reservations. Much of the literature written is based on programs that were temporary in nature and of limited scope. The data is thus limited to short term use of telemedicine.

Literature on telemedicine use in Native Americans is somewhat limited. Though multiple articles were evaluated and synthesized in this literature review, it is vital that further research explore gendered differences in accessing health care, issues with inconsistent access, and computer literacy barriers to telemedicine in Native American populations.

Finally, there is a limited expansion of high-speed Internet to Native American reservations. Because of this, the expansion of Telemedicine is limited due to the high rate of bandwidth that the modality requires.

## Conclusion

This study examined the use of telemedicine in Native Americans, broken down by cost, quality, and access, which are the three central themes of the Affordable Care Act. Our research questions centered around the impact of telemedicine, potential solutions, and improvements to cost, quality, and access for Native American rural communities. Currently, costs of healthcare for indigenous communities in the United States are incurred from travel, time, and social costs such as mismanaging of physical or emotional pain. The quality of such care, when received, is not always patient-centered or culturally competent. Each of these factors impact how accessible healthcare is for Native communities. In an effort to address these problems and draw on the success of temporary programs, innovative solutions for bringing telemedicine to reservations should include reimbursement changes, portable systems, patient and provider education, technological infrastructure, and culturally competent models of care. These solutions advance the goals of the Affordable Care Act and, while limitations to this review do exist, it is evident that the use of telemedicine in rural Native American communities presents a viable option for decreasing cost, increasing quality, and increasing access to healthcare in even the remotest of locations.
